# Supplementary material for: Dynamic changes in anti-SARS-CoV-2 antibodies during SARS-CoV-2 infection and recovery from COVID-19
Source: Nat Commun. 2020 Nov 27;11:6044. doi: 10.1038/s41467-020-19943-y (PMC7699636; doi:10.1038/s41467-020-19943-y)
Supplement: Supplementary file 1 — Supplementary Information [file 41467_2020_19943_MOESM1_ESM.pdf]

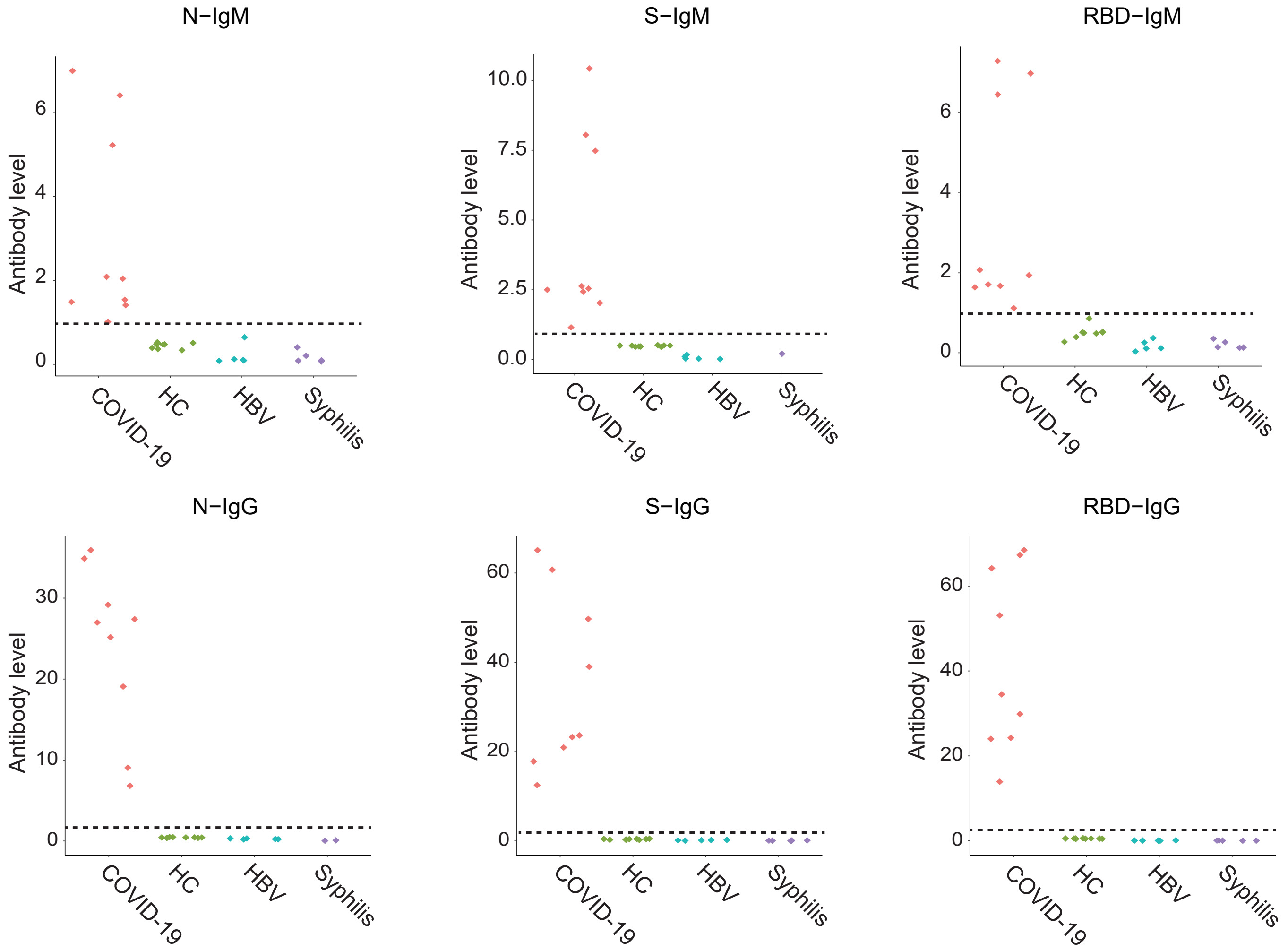

Supplementary Figure 1. The antibody levels of serum samples from COVID-19 patients, healthy controls, patients infected with hepatitis B virus and treponema pallidum.

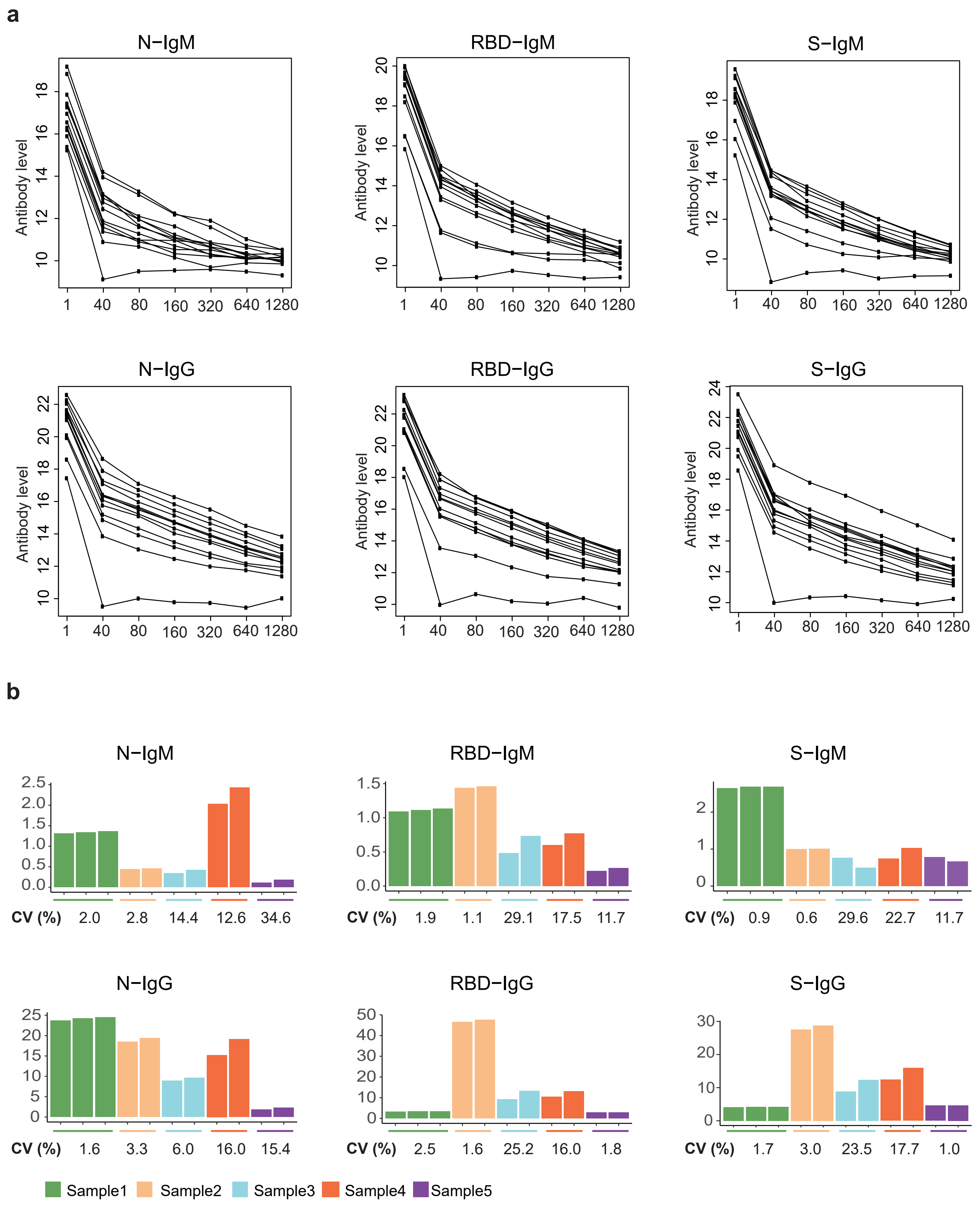

Supplementary Figure 2. The performance validation of antibody detection. (a) The antibody levels of serum samples with different dilutions. (b) Technical replicates of the antibody detection of five serum samples. The coefficient of variation of each sample is shown.

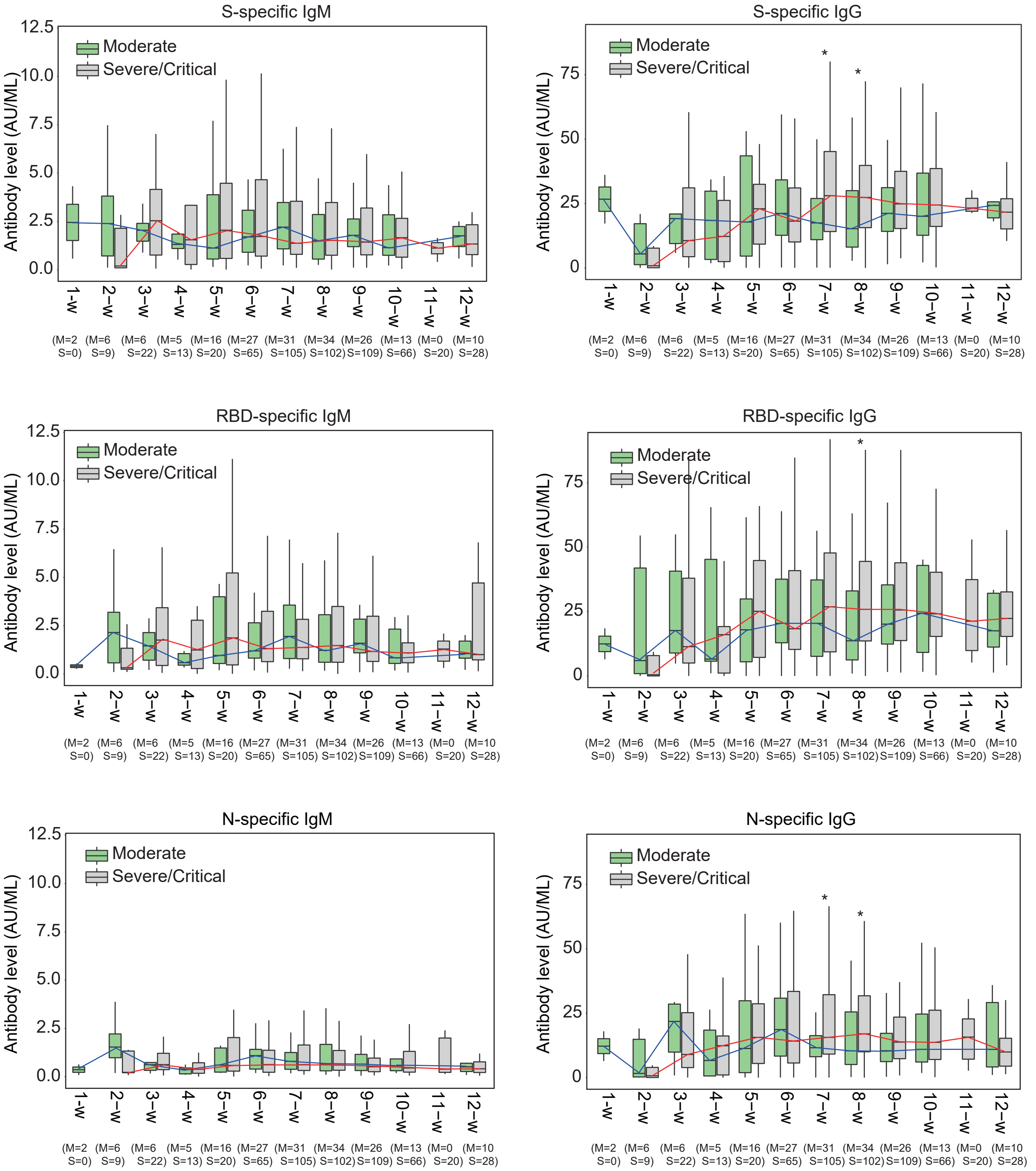

Supplementary Figure 3. The temporal dynamic changes of antibody levels in COVID-19 patients. The dynamics of S-specific, RBD-specific and N-specific IgM and IgG levels of COVID-19 patients. The X-axis displays the weeks since symptom onset, and Y-axis displays the antibody level. Each boxplot depicts the level of antibody in 129 patients with mild/moderate disease, and 289 patients with severe/critical disease. Horizontal lines in the boxplots represent the median, the lower, and the upper hinges correspond to the first and third quartiles, and the whiskers extend from the hinge up to 1.5 times the interquartile range from the hinge. P-values were calculated with two-side Wilcoxon rank-sum test; *P-value < 0.05. The red line based on the median is used to profile the trend among the severe/critical patients, and blue line based on median is used to profile the trend among the mild/moderate patients.

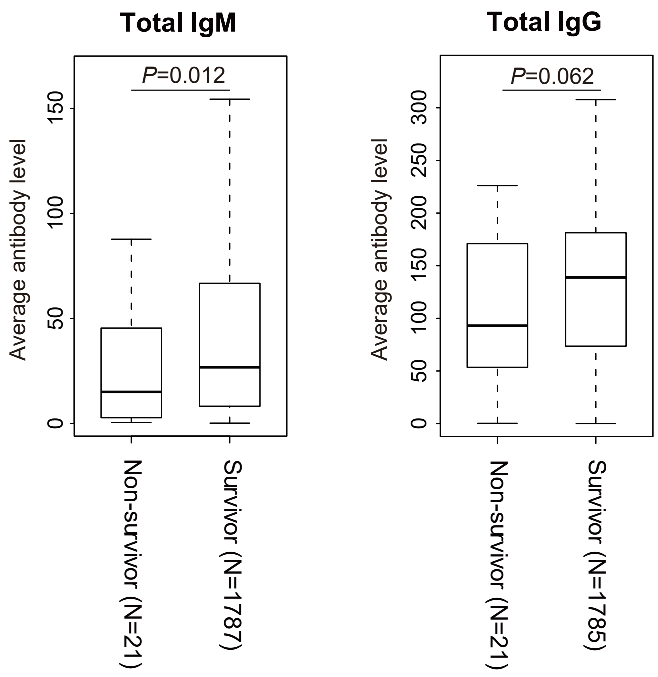

Supplementary Figure 4. The total IgM and IgG levels in COVID-19 survivors and non-survivors. Horizontal lines in the boxplots represent the median, the lower, and the upper hinges correspond to the first and third quartiles, and the whiskers extend from the hinge up to 1.5 times the interquartile range from the hinge. P-values were calculated with two-side Wilcoxon rank-sum test.

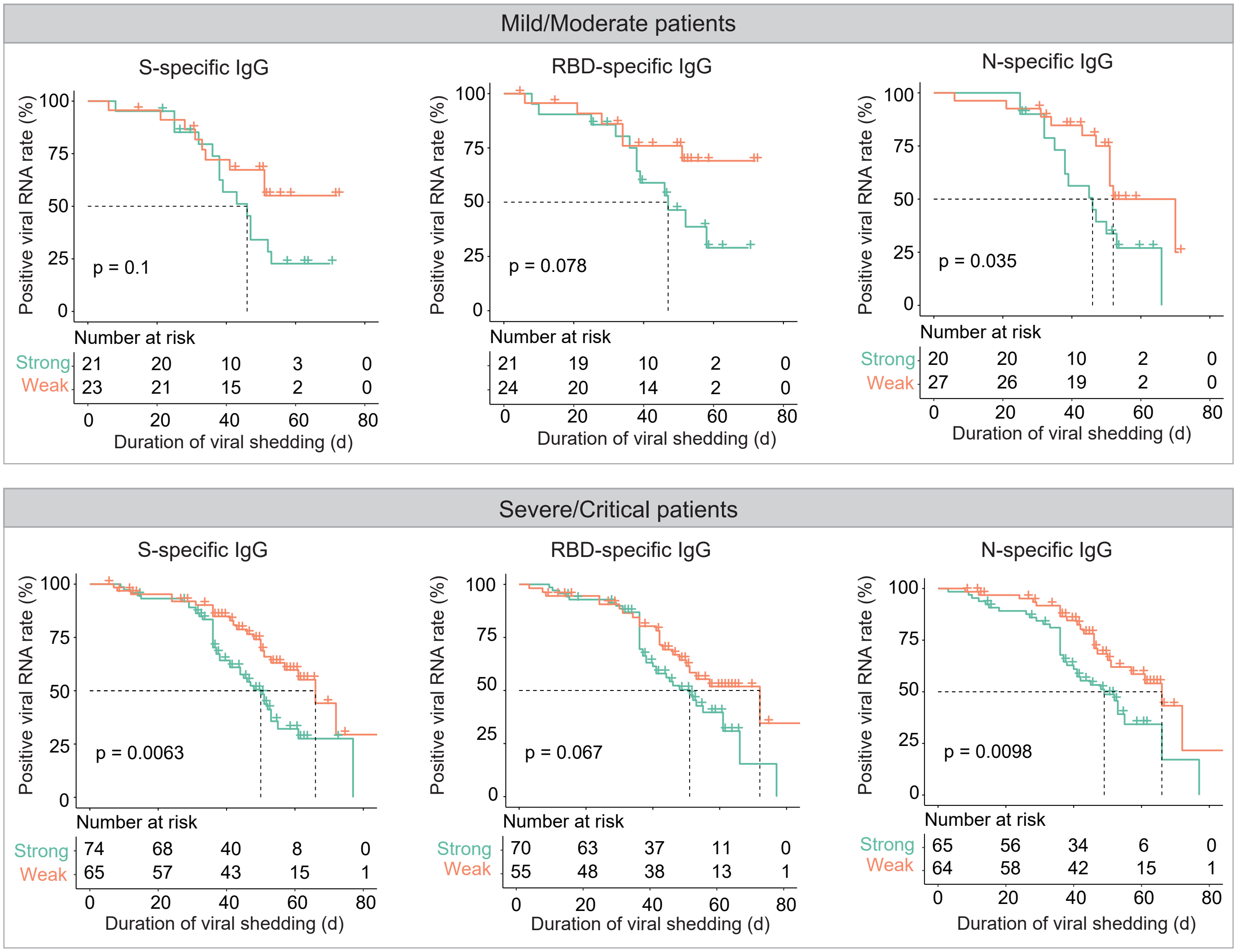

Supplementary Figure 5. The Kaplan–Meier analysis of the viral shedding time in patients with mild/moderate disease and severe/critical disease in the strong antibody response and weak antibody response groups. The X-axis represents the duration of viral shedding (days). The Y-axis represents the proportion of patients with detectable viral RNA. P-values were calculated with log-rank test.

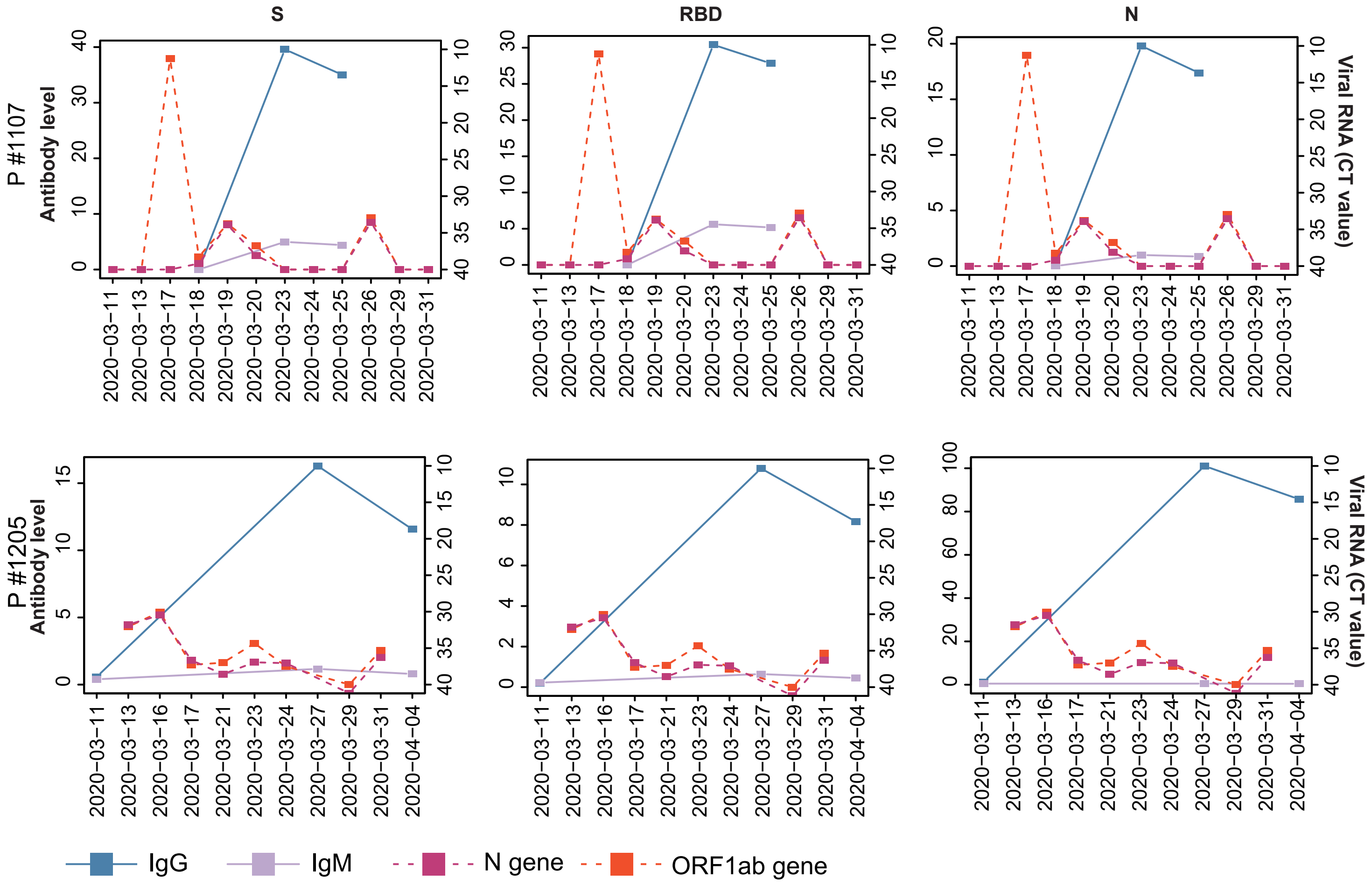

Supplementary Figure 6. The dynamic changes in antibody levels and viral RNA load in Patients #1205 and #1107. The X-axis represents the detection date. The Y-axis on the left represents the antibody level, and the Y-axis on the right represents the CT value of PCR for detection of viral RNA load. CT values <40 were defined as SARS-CoV-2 viral positive. Blue dots represent IgG levels, purple dots represent IgM levels. The ORF1ab and N genes of SARS-CoV-2 are represented as pink and orange dots, respectively.

Supplementary Table 1. The demographic, clinical and laboratory characteristics of this cohort.

	Mild/Moderate (N=795)	Severe/Critical (N=1055)
**Age (yr.)– median (IQR)**	47 (57-65)	55 (64-71.5)
**Sex– no. (%)**		
Female	396 (49.8)	525 (49.8)
Male	399 (50.2)	530 (50.2)
**Comorbidity – no. (%)**		
Hypertension	217 (27.3)	389 (36.9)
Diabetes	107 (13.5)	181 (17.2)
Cardiovascular disease	68 (8.6)	164 (15.5)
Cerebrovascular disease	26 (3.3)	72 (6.8)
Malignancy	15 (1.9)	42 (4)
Chronic obstructive pulmonary disease	28 (3.5)	79 (7.5)
Chronic renal disease	8 (1.0)	23 (2.2)
Chronic liver disease	25 (3.1)	30 (2.8)
Immunodeficiency	2 (0.3)	5 (0.5)
**Days from symptoms onset to admission(d) – median (IQR)**	33 (22-38)	30 (15.8-35)
**Days from admission to discharge(d) – median (IQR)**	11 (7-15)	14 (9-24)
**ICU admission– no. (%)**	0 (0)	63 (6.0)
**Clinical outcomes – no. (%)**		
Discharge from hospital	790 (99.4)	1004 (95.2)
Death	0 (0)	21 (2.0)
Hospitalization	5 (0.6)	30 (2.8)
**Laboratory findings–median (IQR)**		
LYM (%)	29 (23.1-34.3)	23.3 (13.4-30.9)
Mono (%)	7.4 (6.2-8.7)	7.4 (6-9.1)
NEUT (%)	60 (54.1-66.1)	65.2 (56.8-76.8)
LDH (IU/L)	163.4 (144.6-187.1)	201.6 (169.2-252.5)
BNP (pg/mL)	0.01 (0.01-13.59)	28.76 (0.01-130.96)
CRP (mg/L)	1.4 (0.6-3.4)	3.3 (1-15.3)
Urea (mmol/L)	4.5 (3.7-5.5)	5 (4-6.7)
PCT (ng/ml)	0.04 (0.03-0.05)	0.06 (0.04-0.12)
hs-cTnI(ng/ml)	0.01 (0.01-0.01)	0.01 (0.01-0.01)
GLU (mmol/L)	4.8 (4.5-5.5)	5.4 (4.7-7.2)

LYM: lymphocyte; Mono: monocyte; NEUT: neutrophil; LDH: lactate dehydrogenase; BNP: type b natriuretic peptide; CRP: C-reaction protein; Urea: urea nitrogen; PCT: Procalcitonin; hs-cTnI: Hypersensitive cardiac troponin I; GLU: glucose.

Supplementary Table 2. The primers and probes for SARS-CoV-2 real-time RT-PCR assays

**Primer / Probe**	**Sequence (5' to 3')**
ORF1ab-Forward	CCCTGTGGGTTTTACACTTAA
ORF1ab-Reverse	ACGATTGTGCATCAGCTGA
ORF1ab-Probe	FAM-CCGTCTGCGGTATGTGGAAAGGTTATGG-BHQ1
N-Forward	GGGGAACTTCTCCTGCTAGAAT
N-Reverse	CAGACATTTTGCTCTCAAGCTG
N-Probe	ROX-TTGCTGCTGCTTGACAGATT-BHQ2
Rnase P-Forward	AGATTTGGACCTGCGAGCG
Rnase P-Reverse	GAGCGGCTGTCTCCACAAGT
Rnase P-Probe	HEX-TTCTGACCTGAAGGCTCTGCGCG-BHQ1
