# Supplementary material for: Dynamic changes in anti-SARS-CoV-2 antibodies during SARS-CoV-2 infection and recovery from COVID-19
Source: Nat Commun. 2020 Nov 27;11:6044. doi: 10.1038/s41467-020-19943-y (PMC7699636; doi:10.1038/s41467-020-19943-y)
Supplement: Supplementary file 3 — Descriptions of Additional Supplementary Files [file 41467_2020_19943_MOESM3_ESM.pdf]

Descriptions of Additional Supplementary Files

Supplementary Data 1

Description: The number of samples for antibody detection of each patient.

Supplementary Data 2

Description: The raw data of antibody levels of each sample.

Supplementary Data 3

Description: The demographic and clinical characteristics of each patient in this cohort.
