# Supplementary material for: Dynamic changes in anti-SARS-CoV-2 antibodies during SARS-CoV-2 infection and recovery from COVID-19
Source: Nat Commun. 2020 Nov 27;11:6044. doi: 10.1038/s41467-020-19943-y (PMC7699636; doi:10.1038/s41467-020-19943-y)
Supplement: Supplementary file 7 — Reporting Summary [file 41467_2020_19943_MOESM7_ESM.pdf]

nature research

Corresponding author(s): Xinyi Xia
Shukui Wang
Qianghu Wang

Last updated by author(s): Oct 14, 2020

Reporting Summary

Nature Research wishes to improve the reproducibility of the work that we publish. This form provides structure for consistency and transparency in reporting. For further information on Nature Research policies, see our Editorial Policies and the Editorial Policy Checklist.

Statistics

For all statistical analyses, confirm that the following items are present in the figure legend, table legend, main text, or Methods section.

n/a	Confirmed	
☐	☒	The exact sample size (n) for each experimental group/condition, given as a discrete number and unit of measurement
☐	☒	A statement on whether measurements were taken from distinct samples or whether the same sample was measured repeatedly
☐	☒	The statistical test(s) used AND whether they are one- or two-sided<br>*Only common tests should be described solely by name; describe more complex techniques in the Methods section.*
☒	☐	A description of all covariates tested
☒	☐	A description of any assumptions or corrections, such as tests of normality and adjustment for multiple comparisons
☐	☒	A full description of the statistical parameters including central tendency (e.g. means) or other basic estimates (e.g. regression coefficient) AND variation (e.g. standard deviation) or associated estimates of uncertainty (e.g. confidence intervals)
☐	☒	For null hypothesis testing, the test statistic (e.g. F, t, r) with confidence intervals, effect sizes, degrees of freedom and P value noted<br>*Give P values as exact values whenever suitable.*
☒	☐	For Bayesian analysis, information on the choice of priors and Markov chain Monte Carlo settings
☒	☐	For hierarchical and complex designs, identification of the appropriate level for tests and full reporting of outcomes
☒	☐	Estimates of effect sizes (e.g. Cohen's d, Pearson's r), indicating how they were calculated

Our web collection on statistics for biologists contains articles on many of the points above.

Software and code

Policy information about availability of computer code

Data collection	No software was used for data collection.
Data analysis	All analyses were performed using R software (version 3.6.2)

For manuscripts utilizing custom algorithms or software that are central to the research but not yet described in published literature, software must be made available to editors and reviewers. We strongly encourage code deposition in a community repository (e.g. GitHub). See the Nature Research guidelines for submitting code & software for further information.

Data

Policy information about availability of data

All manuscripts must include a data availability statement. This statement should provide the following information, where applicable:

- Accession codes, unique identifiers, or web links for publicly available datasets
- A list of figures that have associated raw data
- A description of any restrictions on data availability

All the data supporting the findings of this study are available within the article and its Supplementary information files. Raw data of clinical information and antibody test of patients are available in Supplementary Data. Source data are provided with this paper.

Field-specific reporting

Life sciences study design

All studies must disclose on these points even when the disclosure is negative.

Sample size	We analyzed the laboratory test results of 1,850 COVID-19 patients. This cohort included 795 mild or moderate cases and 1,055 severe or critical cases. Total SARS-CoV-2 IgM or IgG in the serum were analyzed in 1,850 patients at different time points. 416 of these patients were tested for S-, RBD-, and N- specific IgM and IgG at different time points, including 126 mild or moderate patients and 290 severe or critical patients. No sample size calculation was performed. Sample size was determined/limited by available number of samples in Wuhan Huoshenshan Hospital. The maximum number of samples available was analyzed.
Data exclusions	No data were excluded in this study.
Replication	Our analysis was based on the real-world laboratory test data in emergent pandemic, so replication was not available.
Randomization	This study does not include clinical trial and no randomization was adopted.
Blinding	This study does not include clinical trial and no blinding was adopted.

Reporting for specific materials, systems and methods

We require information from authors about some types of materials, experimental systems and methods used in many studies. Here, indicate whether each material, system or method listed is relevant to your study. If you are not sure if a list item applies to your research, read the appropriate section before selecting a response.

Materials & experimental systems

n/a	Involved in the study	
x		Antibodies
x		Eukaryotic cell lines
x		Palaeontology and archaeology
x		Animals and other organisms
	x	Human research participants
x		Clinical data
x		Dual use research of concern

Methods

n/a	Involved in the study	
x		ChIP-seq
x		Flow cytometry
x		MRI-based neuroimaging

Human research participants

Policy information about studies involving human research participants

Population characteristics	Our dataset included 1,850 COVID-19 patients with a median age of 61±14.4 years, including 929 males and 921 females. This dataset consists of 198 patients younger than 40 years old, 1,008 patients aged 40-65 years old, and 644 patients older than 65 years old.
Recruitment	All of the COVID-19 patients who tested the SARS-CoV-2 specific antibody levels at Wuhan Huoshenshan Hospital were included in our study. No any self-selection of the data was involved.
Ethics oversight	This study was approved by the Medical Ethical Committee of Wuhan Huoshenshan Hospital (HSSLL011), and the Ethical Committee of Nanjing Medical University (2020-511).

Note that full information on the approval of the study protocol must also be provided in the manuscript.
